# Serotonin 5‐HT2C receptor agonism in presymptomatic APP/PS1 mice rescues seizure‐induced premature mortality Perhaps we just plainly state what is happening

**DOI:** 10.1002/alz.087509

**Published:** 2025-01-09

**Authors:** Aaron Del Pozo Sanz, Stephanie Davidson, Melissa Barker‐Haliski

**Affiliations:** ^1^ University of Washington, Seattle, WA USA

## Abstract

**Background:**

Seizures in Alzheimer’s Disease (AD) are increasingly recognized to occur and can increase cognitive decline and reduce survival compared to unaffected age‐matched peers *(Lyou et al. 2018*). Administration of antiseizure medicines (ASMs) to AD patients with epileptiform activity may improve cognition (*Vossel et al. 2021*), yet whether ASMs also beneficially confer long‐term survival benefits in AD is unknown. We have reported that young (presymptomatic) APP/PS1 mice exhibit enhanced chronic focal seizure susceptibility and premature seizure‐induced mortality, which is associated with dysregulated hippocampal serotonin system tone (5‐HT), altered neuroinflammation, and elevated soluble β‐amyloid (Aβ) levels (Del Pozo et al, 2024). We thus hypothesized that lorcaserin, a selective 5‐HT2C receptor agonist, and cannabidiol, a broad‐spectrum anti‐inflammatory and 5‐HT modulating ASM would reduce seizure susceptibility and seizure‐induced excess mortality by attenuating inflammation and normalizing 5‐HT system dysregulation in kindled presymptomatic APP/PS1 mice.

**Methods:**

Male and female 2‐month‐old APP/PS1 and their wild‐type (WT) littermates underwent corneal or sham kindling, a model of chronic focal seizures (Beckman et al. 2020), for 15 days until achieving criterion (N = 8/group/sex). APP/PS1 mice received either lorcaserin [10 mg/kg, (b.i.d.)], cannabidiol [100 mg/kg (q.d.)], or vehicle (VEH) i.p. during kindling. Seizure acquisition and survival were quantified. Kindling‐induced changes in soluble Aβ levels in isolated hippocampus were then measured by western blot. Neuroinflammation was quantified by immunohistochemistry.

**Results:**

Whereas lorcaserin administration in neither male nor female APP/PS1 mice did not affect seizure severity or kindling versus APP/PS1‐VEH, cannabidiol administration blunted female kindling rate. Both male and female APP/PS1 mice treated with lorcaserin exhibited significant improvements in survival. Despite effects on seizure burden in males, an opposite effect of treatment occurred on mortality: only cannabidiol effectively prevented excess seizure‐induced mortality of APP/PS1 mice. The extent of neuroinflammation and other pathological AD hallmarks (soluble Aβ) will be further discussed.

**Conclusion:**

5‐HT2C agonism rescues seizure‐induce premature mortality independently of direct effects on seizure susceptibility in APP/PS1 mice. CBD shows a sex‐dependent effect, only reducing male seizure‐induced mortality. These results support the use of pharmacological reduction of neuronal hyperexcitability as a strategy to modify disease burden and improve long‐term outcomes in AD.

